# Local adaptation to temperature in populations and clonal lineages of the Irish potato famine pathogen *Phytophthora infestans*

**DOI:** 10.1002/ece3.2282

**Published:** 2016-08-14

**Authors:** Nicolas Mariette, Annabelle Androdias, Romain Mabon, Roselyne Corbière, Bruno Marquer, Josselin Montarry, Didier Andrivon

**Affiliations:** ^1^ INRAUMR IGEPP (Institute for Genetics, Environment and Plant Protection)35653Le Rheu CedexFrance

**Keywords:** Climate change, epidemic, late blight, life‐history traits, phenotypic plasticity, plant pathogen, temperature adaptation

## Abstract

Environmental factors such as temperature strongly impact microbial communities. In the current context of global warming, it is therefore crucial to understand the effects of these factors on human, animal, or plant pathogens. Here, we used a common‐garden experiment to analyze the thermal responses of three life‐history traits (latent period, lesion growth, spore number) in isolates of the potato late blight pathogen *Phytophthora infestans* from different climatic zones. We also used a fitness index (FI) aggregating these traits into a single parameter. The experiments revealed patterns of local adaptation to temperature for several traits and for the FI, both between populations and within clonal lineages. Local adaptation to temperature could result from selection for increased survival between epidemics, when isolates are exposed to more extreme climatic conditions than during epidemics. We also showed different thermal responses among two clonal lineages sympatric in western Europe, with lower performances of lineage 13_A2 compared to 6_A1, especially at low temperatures. These data therefore stress the importance of thermal adaptation in a widespread, invasive pathogen, where adaptation is usually considered almost exclusively with respect to host plants. This must now be taken into account to explain, and possibly predict, the global distribution of specific lineages and their epidemic potential.

## Introduction

Temperature is a major abiotic factor impacting all levels of biological functions, from molecules to ecosystems (Hochachka and Somero 2002); as such, it influences all ecological communities and interactions (Cossins and Bowler 1987). In host–pathogen interactions, temperature can strongly affect epidemic development, notably by acting on key stages of the pathogen life cycle (Tooley et al. 2009; Sharma et al. 2011). In the context of climate change, it is crucial to analyze and predict patterns of evolutionary adaptation of agronomically or medically relevant pathogens to different thermal environments (Bennett and Lenski 1999; Mboup et al. 2012). Indeed, the Earth's climate has warmed at an unprecedented rate since the last century, with a rise of global surface temperature by 0.74°C on average. This phenomenon seems to continue, and the temperature increase could reach up to 4°C by the end of the 21st century according to the most recent projections (IPCC 2014).

Climate change will probably modify ecological niches as well as the phenology and geographical ranges of species, leading to unavoidable modifications in the relationships between species (see reviews by Parmesan 2006; Bellard et al. 2012). The fast elevation of temperatures is also expected to impact intraspecific interactions. For instance, the genetic composition of a local population may change due to the selection of genotypes performing better in the new climatic conditions (i.e., higher temperatures), as suggested by Hoffmann and Sgrò (2011). Local populations may also have to cope with warm‐adapted genotypes migrating from lower latitudes (van Doorslaer et al. 2009; Bebber et al. 2013). The risk of such a displacement of the resident gene pool will depend upon the extent to which individual populations within a species are locally adapted to prevailing temperatures (Mitchell and Lampert 2000). Testing local adaptation in parasites should best rely on comparisons between current (and presumably adapted) populations and their (presumably less adapted) ancestors. Unfortunately, such comparisons are normally impossible, as we no longer have access to ancient populations. This imposes that of local adaptive patterns are generally tested in common‐garden experiments (Lively 1996; Kaltz and Shykoff 1998), where either the performances of different populations within environments (the “local vs. foreign” criterion) or the performances of a given population across environments (the “home vs. away” criterion) are compared (Kawecki and Ebert 2004; Blanquart et al. 2013).

In microorganisms, local adaptation to environmental factors such as salinity or pH has been reported many times (e.g., Weisse et al. 2011; Rengefors et al. 2015). Some studies showing local adaptive patterns to temperature are also present in the literature. For example, local thermal adaptation in the “*Spumella*‐like” flagellates has been shown in an experiment including strains collected in warm, temperate, and Antarctic regions (Boenigk et al. 2007). Similarly, different thermal adaptation patterns were observed in Northern and Southern French isolates of *Puccinia striiformis*, the fungal pathogen causing stripe rust on wheat (Mboup et al. 2012). Zhan and McDonald (2011) also found evidence of local adaptation to temperature in another fungal pathogen of wheat, *Mycosphaerella graminicola*, when comparing growth rates of isolates sampled across five continents. However, in most of these studies, adaptation results in a genetic differentiation of isolates/pathotypes according to climatic zones (Mboup et al. 2012). We are not aware of reports of thermal adaptation within clonal lineages of pathogens, although many parasites multiply primarily asexually.

Pathogens of annual crops are generally found in agroecosystems distributed across a wide range of climates, providing good model systems for the study of local adaptation to temperature at different geographical and genetic scales (Stefansson et al. 2013). This is for instance the case of *Phytophthora infestans*, which causes late blight, a serious threat to potato (*Solanum tuberosum*) and tomato (*Solanum lypersicum*) production throughout the world (Kamoun et al. 2015). Its life cycle is primarily aerial, with polycyclic epidemics in which asexual sporangia containing infective zoospores are dispersed from host to host by water or wind, potentially over long distances (Aylor 2003; Glais et al. 2014). *Phytophthora infestans* is a heterothallic organism with two mating types, A1 and A2. Between crop seasons, this pathogen can therefore survive as asexual clones in potato tubers or *via* the long‐lived oospores resulting from the sexual cycle wherever the two mating types coexist and mating occurs (Drenth et al. 1995; Andrivon et al. 2013). Originally from central Mexico (Goss et al. 2014), *P. infestans* can be found wherever potato is grown, in a wide range of climates. This is the case in Europe, where the pathogen is present from Scandinavia to the Mediterranean Basin (MB). The recent development of polymorphic microsatellite markers (Lees et al. 2006; Li et al. 2013a) allowed to reveal marked differences in the genetic structure of *P. infestans* populations between these areas. Populations from northern Europe (NE) display high levels of genetic diversity, a consequence of sexual reproduction (Sjöholm et al. 2013; Runno‐Paurson et al. 2016). By contrast, and despite the coexistence of both mating types, *P. infestans* populations from western Europe (WE) and the MB are largely clonal and dominated by a few genotypes (Montarry et al. 2010a; Gisi et al. 2011; Cooke et al. 2012; Harbaoui et al. 2014; Mariette et al. 2016). During the past few years, two clonal lineages, 13_A2 and 6_A1, have been reported at high frequencies in west European populations (Cooke et al. 2012; Mariette et al. 2016). Isolates belonging to the 13_A2 clonal lineage are also found in other areas, such as the MB or Asia (Corbière et al. 2010; Li et al. 2013b; Chowdappa et al. 2015).

Here, we conducted a common‐garden experiment in which 42 *P. infestans* isolates from different climatic areas were exposed to four temperatures (10, 14, 18, and 24°C). Three life‐history traits were assessed, allowing the computation of a fitness index (FI) of derived from the basic reproduction number (Montarry et al. 2010b). Thanks to this experimental design, we tested, for the first time in this species, the hypothesis of a local adaptation to temperature in *P. infestans*. The originality of this study also resides on the fact that we also tested whether isolates belonging to a same clonal lineage but sampled in different climatic areas showed specific local adaptive patterns to temperature. This aspect is therefore generally not treated in this type of studies. The third objective of our work was to provide insights into the specific thermal sensitivity of European *P. infestans* clonal lineages, for which little information is available so far.

## Materials and Methods

### Origin of *P. infestans* isolates

One hundred and ninety‐nine *P. infestans* isolates were sampled in potato fields located in seven countries among three geographical areas: 45 isolates from NE (Denmark, Estonia, Latvia and Lithuania), 137 from WE (Brittany, France), and 17 from MB (Algeria and Cyprus) (Fig. 4; Table S1). The sampling took place during the mid‐ and end stages of late blight epidemics, from May to September 2013 depending on the area. The three geographical areas have been chosen for their climatic specificities, particularly temperatures encountered along the year. Northern Europe features severe winters and relatively brief, rather cool summers, whereas in WE, the annual temperature range is relatively narrow with cool winters and summers. For its part, the climate of the MB is characterized by mild winters and hot, dry summers. To describe the local thermal conditions, we used climatic variables available on the Climate-Data.org (http://climate-data.org); monthly temperature means were used to estimate annual mean, variance, minimum, and maximum of each sampling area (Table S1).

Isolates were obtained from potato leaflets with single lesions (only one leaf per plant was taken), by placing a fragment of infected leaf tissue on tuber slices of a susceptible potato cultivar (Bintje, Spunta, or Berber). After incubation for 5–7 days at 15–18°C in growth chambers, pure cultures were established by transferring the hyphal mats growing on top of the slices to pea or Rye B agar media supplemented with antibiotics (ampicillin, rifamycin, pimaricin). After about 10 days, growing colonies were transferred to agar media without antibiotics, and subsequently maintained at 15°C in darkness.

### Genetic characterization and selection of isolates for the experiments

**Figure 1:**
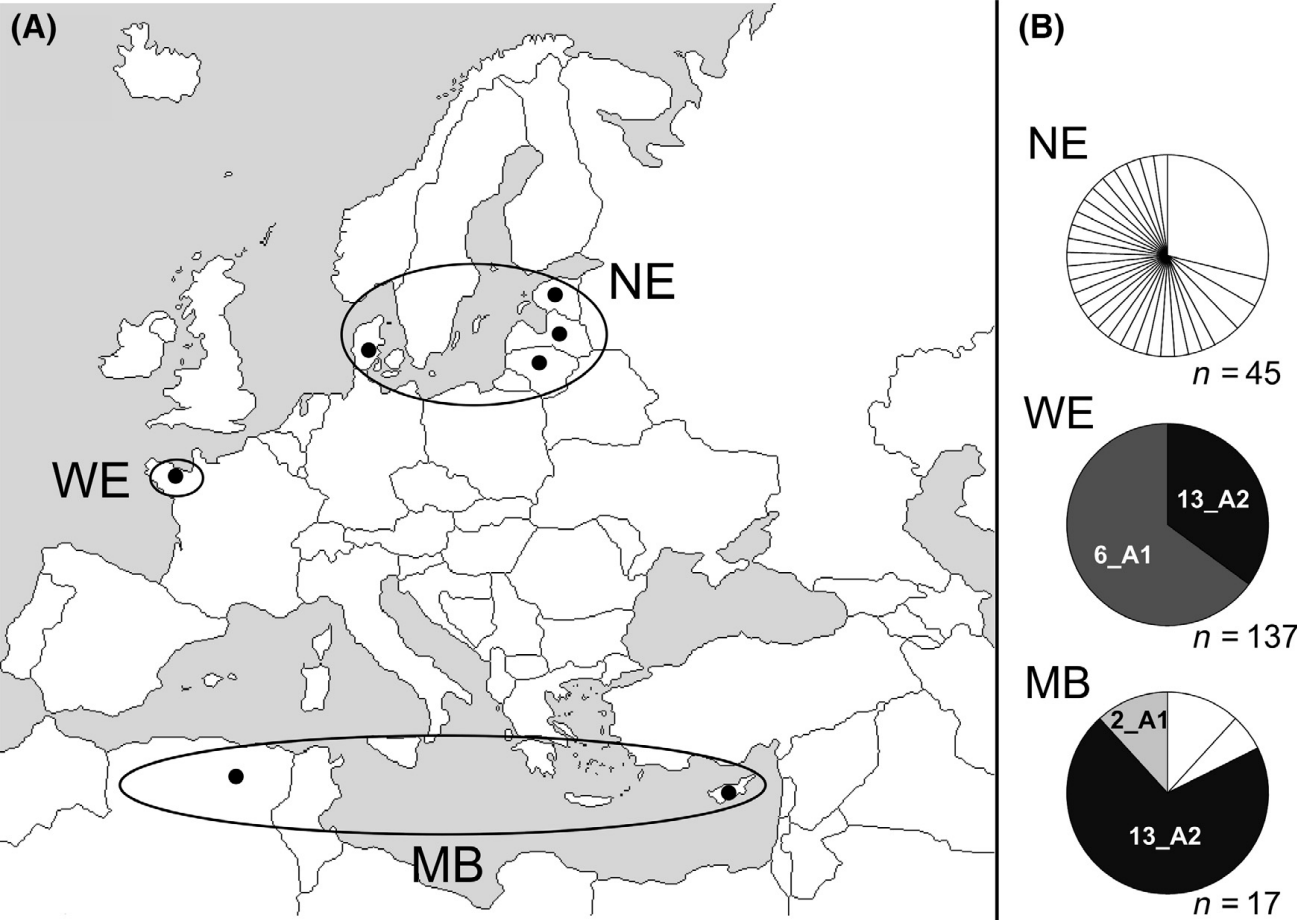
Sampling characteristics with the location of source *Phytophthora infestans* populations (A) and clonal lineage distribution among the populations (B). Populations are coded as follows: NE, northern Europe; WE, western Europe; MB, Mediterranean Basin. The seven points on the map represent the main location of potato fields sampled in each country. Unknown genotypes are represented in white in the graphics.

All the 199 *P. infestans* isolates have been characterized both for their mating type through pairing tests (Montarry et al. 2008) and their genotype using 17 polymorphic microsatellites markers: Pi02, PiG11, Pi4B, Pi4G (Knapova and Gisi 2002), D13, Pi04, Pi16, Pi33, Pi56, Pi63, Pi70 (Lees et al. 2006), PinfSSR2, PinfSSR4, PinfSSR6a, PinfSSR7, PinfSSR8, and PinfSSR11 (Li et al. 2013a). This genetic characterization allowed to identify the multilocus genotypes (MLGs) and the corresponding clonal lineages among the 199 isolates (Mariette et al. 2016; Fig. 1). From this characterization, we have selected 42 isolates representing as faithfully as possible the genetic diversity of each area: 16 sampled in NE, 17 in WE, and nine in the MB (Fig. 1; Table S2). Besides, this selection of isolates has allowed treating our three research questions: Firstly, all the 42 isolates were used to test the hypothesis of a local thermal adaptation as we compared the temperature responses of isolates from the three geographic areas, regardless of the genotype characteristics, and secondly, fourteen 13_A2 isolates from both WE and the MB (eight from WE and six from MB) were therefore included in the selection with the aim to test the local adaptation hypothesis within a clonal lineage. Indeed, a same clonal lineage was never observed in all three areas, but isolates belonging to the 13_A2 clonal lineage have been sampled in these two areas. Thirdly, nine west European isolates belonging to the 6_A1 clonal lineage were also selected with the aim to compare their pattern responses with those of the west European 13_A2 isolates.

### Temperature response experiments

Three life‐history traits linked to aggressiveness of the *P. infestans* isolates were measured in a range of four temperatures (10, 14, 18, and 24°C) using a common‐garden experiment. This temperature range was chosen because it covers the biological activity of *P. infestans* (Mizubuti and Fry 1998; Maziero et al. 2009).

Aggressiveness tests were performed on detached leaflets of the potato cultivar Bintje, susceptible to late blight. Plants were grown from certified seed tubers in 13‐cm pots (one tuber per pot) filled with 1:1:1 sand–peat–compost mixture placed in a glasshouse regulated at 15–20°C (night/day temperatures) with 16 h of photoperiod. Once a week, plants were watered with a nutrient solution (Hakaphos NPK 15/10/15, Hakaphos Blau, Compo France SAS, Roche les Beaupré, France). For the inoculum preparation and aggressiveness tests, leaflets of similar size were picked from the median area of 6‐ to 8‐week‐old plants.

Before their aggressiveness assessment, isolates were multiplied separately on detached leaflets of cv. Bintje in order to restore pathogenicity possibly lost during axenic cultures (Jinks and Grindle 1963). To this end, droplets of sporangia suspensions prepared from 3‐ to 4‐week‐old pea agar cultures (by flooding with sterile water and scrapping the colony surface) were deposited on the underside of detached leaflets. After seven to 10 days of incubation in humid chambers under controlled conditions (18°C with 16 h of day length), newly formed sporangia were collected from the leaflets in sterile water. Sporangia were counted using a hemocytometer and diluted to a concentration of 5 × 10^4^ sporangia per mL. Finally, before inoculation for aggressiveness determination, the suspensions were kept at 4°C for approximately 2–3 h to promote zoospore release.

Each isolate was inoculated separately onto 24 detached leaflets of cv. Bintje by depositing a 20 μL droplet of the prepared sporangial suspensions (about 1000 sporangia) on the center of each leaflet. Before inoculation, leaflets were placed in pairs, abaxial face up, onto the lids of inverted Petri dishes containing 10 g·L^−1^ water agar and acting as humid chambers. Six leaflets (i.e., three dishes) were kept in clear boxes and incubated in climatic chambers regulated at each temperature tested (i.e., 10, 14, 18, and 24°C) with 16 h of day length. All incubations were performed in the same climatic chambers, and the temperature prevailing in each climatic chamber was recorded every hour using Thermo Tracer recorders (Oceasoft, Montpellier, France). This tracking has revealed that the temperature increased by 2°C during the diurnal period because of the heat generated by the lights. As a consequence, the diurnal temperatures within the chambers regulated at 10, 14, 18, and 24°C actually were 12, 16, 20, and 26°C, respectively (Fig. S1).

Three aggressiveness components were measured: the latent period (LP), the sporulating lesion growth rate (LGR), and the sporangia production (SP). The LP, namely the elapsed time between inoculation and first sporulation, was assessed by observing daily the leaflets under a magnifying glass to check the appearance of sporangia. For each leaflet, 3 days after the observation of the first sporangia, we measured the two diameters (one along the midrib and one perpendicular to it) of the sporulating lesion expansion. The lesion area was then calculated from these measurements assuming an elliptic shape. The sporulating LGR (LGR), expressed in mm²·day^−1^, was calculated by dividing the sporulating lesion area by three (i.e., the number of days since the onset of sporulation). Immediately after measuring the lesion diameters, sporangia were washed from leaflets in 10 mL of Isoton II (saline buffer; Beckman Coulter, Villepinte, France). Suspensions were kept in glass tubes at −20°C until the counting of the sporangia with a Coulter Z2 counter (Beckman Coulter) equipped with a 100‐μm aperture tube, allowing the determination of the number of sporangia produced on the lesion, that is the SP. We calculated the FI as described by Montarry et al. (2010b), setting the mortality parameter (*μ*) at 10 days, that is, the maximal experiment duration over the range of temperatures tested. The experiment was repeated twice, between March and May 2014. In order to minimize experimental errors, all inoculations and measurements were performed by a limited number of skilled operators.

### Statistical analyses

All statistical analyses were carried out using the software R, version 3.1.0 (R Core Team, 2014), and the significance threshold was fixed at *α *= 0.05. We compared thermal responses of *P. infestans* isolates at three levels: (1) among isolates coming from the three geographical areas, (2) between the 13_A2 isolates collected in two geographical areas (WE and MB), and (3) between the 13_A2 and 6_A1 isolates sampled in WE. Normality and homogeneity of variances were checked with the Shapiro–Wilk and the Levene's tests, respectively. For each level of comparison, the effects of temperature and origin (geographical or clonal) on LP were tested using the rank‐sum tests of Kruskal–Wallis and Wilcoxon because assumptions of homoskedasticity and normal distribution were not met to use parametric tests. The impact of temperature, origin (geographical or clonal), and their interactions on LGR, SP, and FI were assessed with linear mixed models using the “lme4” package, version 3.0.3 (Bates et al. 2011). In these models, temperature, population origin (geographical or clonal), and test repetition were treated as fixed factors and the MLG was rated as random factor. SP was square‐root‐transformed to satisfy the assumptions of homoskedasticity and normality. The effects of temperature, origin (geographical or clonal), and the interactions temperature × origin and temperature × origin × test repetition were tested with a Wald's test. When needed, pairwise comparisons of least square means (LSMeans; the “lsmeans” package version 3.0.3) have been performed using the Tukey's method for *P*‐values adjustment.

## Results

### Temperature responses among populations from the three climate zones

The general shape of thermal reaction norms for LP, LGR, SP, and FI was consistent with expectations in all isolates, regardless of their geographical origin: low performances at 10°C, an increase in performance with temperature up to a maximum at 18°C, and then a decline at 24°C, especially for SP and FI (Table 1).

**Table 1:** Aggressiveness components of the 42 *Phytophthora infestans* isolates sampled in the three geographical areas (northern Europe, western Europe, and Mediterranean Basin)

Variable	Temp. (°C)	Value/populations^1^		
		Northern Europe (*n* = 16)	Western Europe (*n* = 17)	Mediterranean Basin (*n* = 9)
LP	10	5.51 (0.08)a	5.11 (0.05)a	5.23 (0.08)a
	14	4.10 (0.06)b	3.78 (0.04)b	4.01 (0.06)b
	18	3.09 (0.04)d	2.93 (0.03)c	3.05 (0.03)c
	24	3.29 (0.05)c	2.95 (0.03)c	2.99 (0.07)c
LGR	10	260.6 (7.9)d	298.4 (7.0)c	240.1 (7.3)c
	14	399.9 (8.2)c	468.4 (7.9)b	393.2 (9.5)b
	18	602.2 (8.3)a	647.1 (8.3)a	634.6 (11.1)a
	24	549.7 (11.7)b	622.2 (9.0)a	590.9 (11.4)a
SP	10	105,181 (5169)c	95,092 (3977)c	82,916 (4457)c
	14	188,760 (8299)b	216,823 (7354)b	168,150 (9100)b
	18	302,369 (12,483)a	375,911 (11,118)a	388,046 (14,792)a
	24	57,733 (4671)d	84,054 (4131)c	78,888 (4897)c
FI	10	88,314 (3432)b	75,186 (2610)b	74,570 (3267)b
	14	134,299 (5430)c	140,330 (3461)c	121,044 (5217)c
	18	176,647 (7133)d	209,536 (5428)d	220,017 (5428)d
	24	35,187 (2588)a	47,907 (2204)a	46,673 (2608)a

LP, latent period (days); LGR, lesion growth rate (mm²·day^−1^); SP, sporangia production (no. sporangia/lesion); FI, fitness index.

^1^Mean (±standard error); Different letters beside the values indicate significant differences between the temperatures within each geographic area at *P *<* *0.05 (Wilcoxon rank‐sum tests or lsmeans).

**Figure 2:**
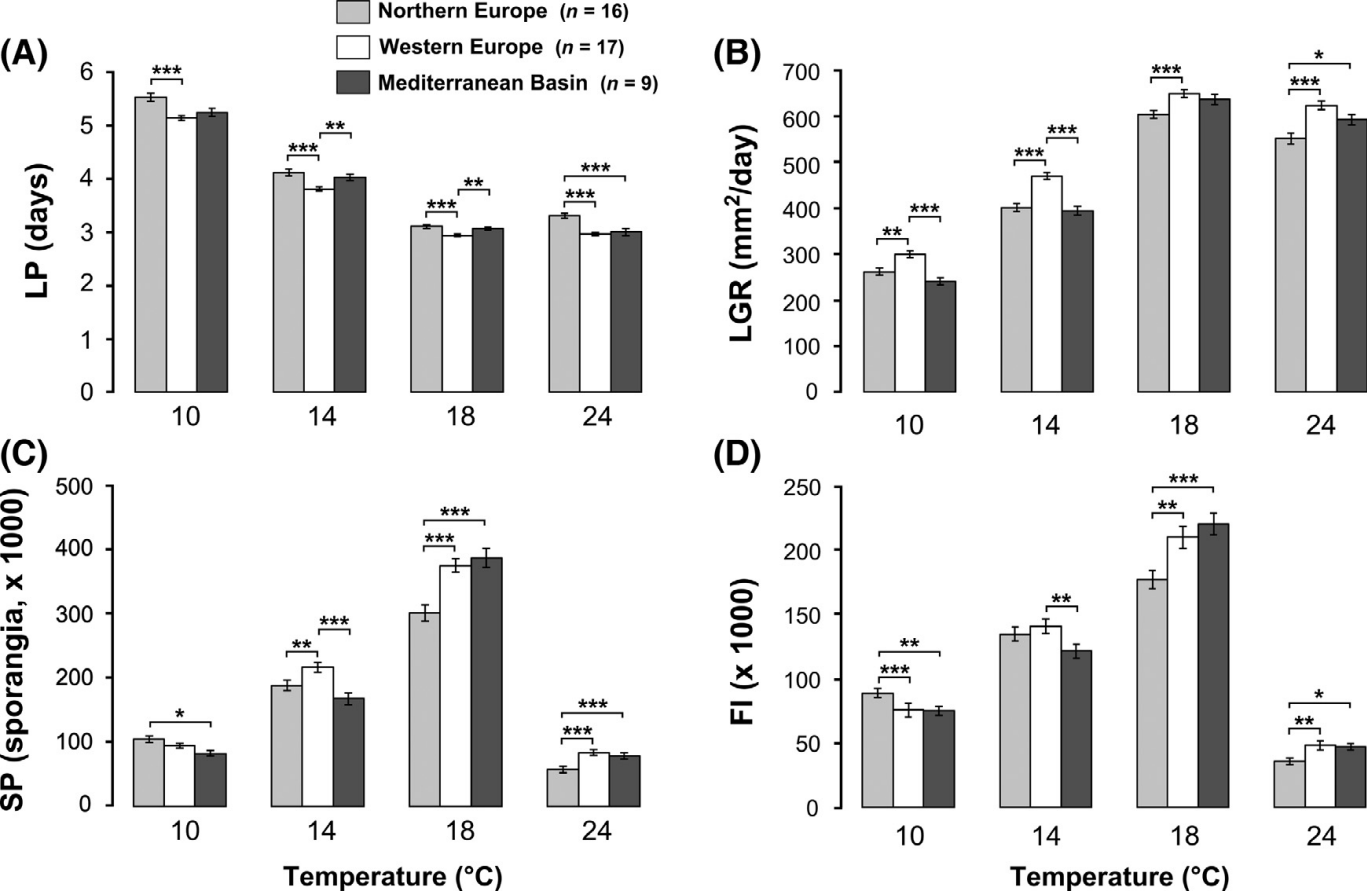
Temperature responses of 42 *Phytophthora infestans* isolates sampled in three geographic areas for the latent period (A), the lesion growth rate (B), the sporangia production (C), and the sporangia size (D) (mean ± SE). Significant differences between the geographical areas at a given temperature, as revealed by Wilcoxon rank‐sum tests or lsmeans: *P <* *0.05, **P <* *0.01, ***P <* *0.001.

Despite the similar reaction norms of *P. infestans* isolates, differences in temperature responses were detected between the three geographic areas (Fig. 2; Table 2). Nordic isolates had long LPs at the four temperatures tested (Fig. 2A). At 24°C, their LP was significantly longer (i.e., 3.3 days) than that of isolates sampled in the other two areas (i.e., 2.9 days; Wilcoxon rank‐sum tests, *P *<* *0.001), whereas no significant differences between Nordic and Mediterranean isolates were detected at the other temperatures (Wilcoxon rank‐sum tests, *P *=* *0.9). West European isolates showed the highest LGRs over the whole temperature range (Fig. 2B). They grew significantly faster than Nordic isolates at the four temperatures tested (Wald's test, lsmeans post hoc comparison; *P *<* *0.01–<0.001) and then Mediterranean isolates at 10 and 14°C (Wald's test, lsmeans post hoc comparison; *P *<* *0.001). Moreover, if no significant difference was detected between isolates from NE and MB at low temperatures (Wald's test, lsmeans post hoc comparison; *P *>* *0.05), the Nordic isolates had a lower LGR at high temperatures (Fig. 2B). At 18°C, the difference was just above the significance threshold (Wald's test, lsmeans post hoc comparison; *P *=* *0.06) and was significant at 24°C (Wald's test, lsmeans post hoc comparison; *P *=* *0.014). At 10°C, the Nordic isolates had the highest SP with 10.5 × 10^4^ spores/lesion, significantly higher than the Mediterranean isolates (i.e., 8.3 × 10^4^ spores/lesion; Wald's test, lsmeans post hoc comparison; *P *=* *0.049; Fig. 2C). The opposite was true at high temperatures (18 and 24°C), explaining the significant geographic origin × temperature interaction (Table 2). At 14°C, the west European isolates had the highest SP (Wald's test, lsmeans post hoc comparison; *P *<* *0.01–<0.001), whereas no difference was observed between isolates from the two other areas (Wald's test, lsmeans post hoc comparison; *P *=* *0.35). As for SP, the geographic origin × temperature interaction was significant for the FI (Table 2). Indeed, if the Nordic isolates had a significant highest FI than other isolates at 10°C, the opposite was observed at 18°C and 24°C (lsmeans post hoc comparison; *P *<* *0.05–<0.001; Fig. 2D). Finally, at 14°C, the west European isolates had the highest FI, even if no significant difference was found with the Nordic isolates (Wald's test, lsmeans post hoc comparison; *P *=* *0.22).

**Table 2:** Linear mixed‐model analysis of the geographic origin effect (northern Europe, western Europe, and Mediterranean Basin) and the temperature effect (10, 14, 18, and 24°C) on the lesion growth rate (LGR), the sporangia production (SP), and the fitness index (FI) of 42 *Phytophthora infestans* isolates

Response variable	LGR			SP			FI		
	df	Wald *χ*²	*P *> *χ*²	df	Wald *χ*²	*P *> *χ*²	df	Wald *χ*²	*P *> *χ*²
Origin	2	6.250	0.0439*	2	1.878	0.3909	2	1.252	0.5347
Temperature	3	1290.494	<0.0001***	3	961.517	<0.0001***	3	946.802	<0.0001***
Origin × temperature	6	9.909	0.1285	6	23.733	<0.0001***	6	24.781	<0.0001***
Origin × temperature × repetition	12	16.169	0.1836	12	11.028	0.5265	12	11.890	0.4545

Significance levels as follows: *P <* *0.05, ***P <* *0.001.

### Intraclonal adaptation to temperature within the 13_A2 clonal lineage

**Figure 3:**
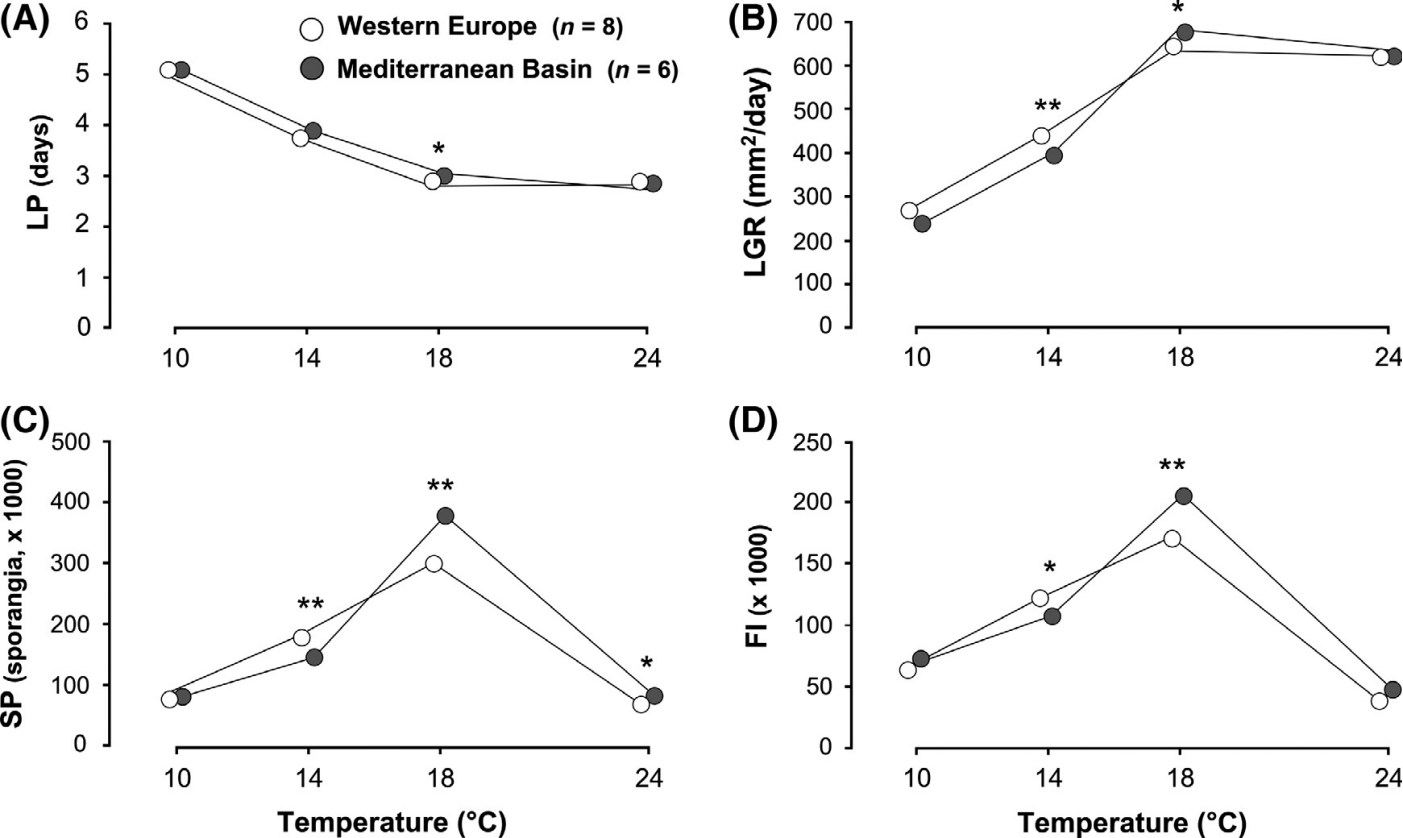
Temperature responses of 14 *Phytophthora infestans* isolates belonging to the 13_A2 clonal lineage sampled in two geographical areas for latent period (A), lesion growth rate (B), sporangia production (C), and sporangia size (D). SE were omitted for clarity. Significant differences between the geographical areas at a given temperature, as revealed by Wilcoxon rank‐sum tests or lsmeans: *P <* *0.05, **P <* *0.01.

The LPs were generally similar among the 14 isolates tested belonging to the 13_A2 clonal lineage but coming from different geographic areas (WE and MB). This was true over the whole temperature range, except at 18°C where west European isolates had a significantly shorter LP (Wilcoxon rank‐sum tests, *P *=* *0.041; Fig. 3A). Significant effects of origin × temperature interaction were observed for LGR, SP, and FI (Table 3). Indeed, west European isolates grew faster than Mediterranean isolates at low temperatures (10 and 14°C; Fig. 3B), whereas Mediterranean isolates performed better at high temperatures, especially at 18°C (Wald's test, lsmeans post hoc comparison; *P *=* *0.049; Fig. 3B). These temperature‐dependent differences were also observed for SP with more sporangia produced by the west European isolates at 14°C compared to the Mediterranean isolates (Wald's test, lsmeans post hoc comparison; *P *=* *0.007; Fig. 3C), while the opposite was observed at 18 and 24°C (Wald's test, lsmeans post hoc comparison; *P *<* *0.05–<0.01; Fig. 3C). Finally, west European isolates had a significantly higher FI than Mediterranean isolates at 14°C, while the reverse trend was found at 18°C (Wald's test, lsmeans post hoc comparison; *P *<* *0.05–<0.01; Fig. 3D).

**Table 3:** Linear mixed‐model analysis of the geographic origin (western Europe and Mediterranean Basin) and the temperature effect (10, 14, 18, and 24°C) on the lesion growth rate (LGR), the sporangia production (SP), and the fitness index (FI) of 14 *Phytophthora infestans* isolates belonging to the 13_A2 clonal lineage

Response variable	LGR			SP			FI		
	df	Wald *χ*²	*P *> *χ*²	df	Wald *χ*²	*P *> *χ*²	df	Wald *χ*²	*P *> *χ*²
Origin	1	0.803	0.3703	1	0.517	0.4723	1	1.249	0.2637
Temperature	3	870.939	<0.0001***	3	600.740	<0.0001***	3	561.702	<0.0001***
Origin × temperature	3	7.923	0.0476*	3	13.807	0.0032**	3	9.453	0.0238*
Origin × temperature × repetition	8	8.834	0.3565	8	7.662	0.4671	8	6.174	0.6278

Significance levels as follows: *P <* *0.05, **P <* *0.01, ***P <* *0.001.

### Adaptation patterns to temperature in sympatric clones: 13_A2 and 6_A1 isolates from WE

**Figure 4:**
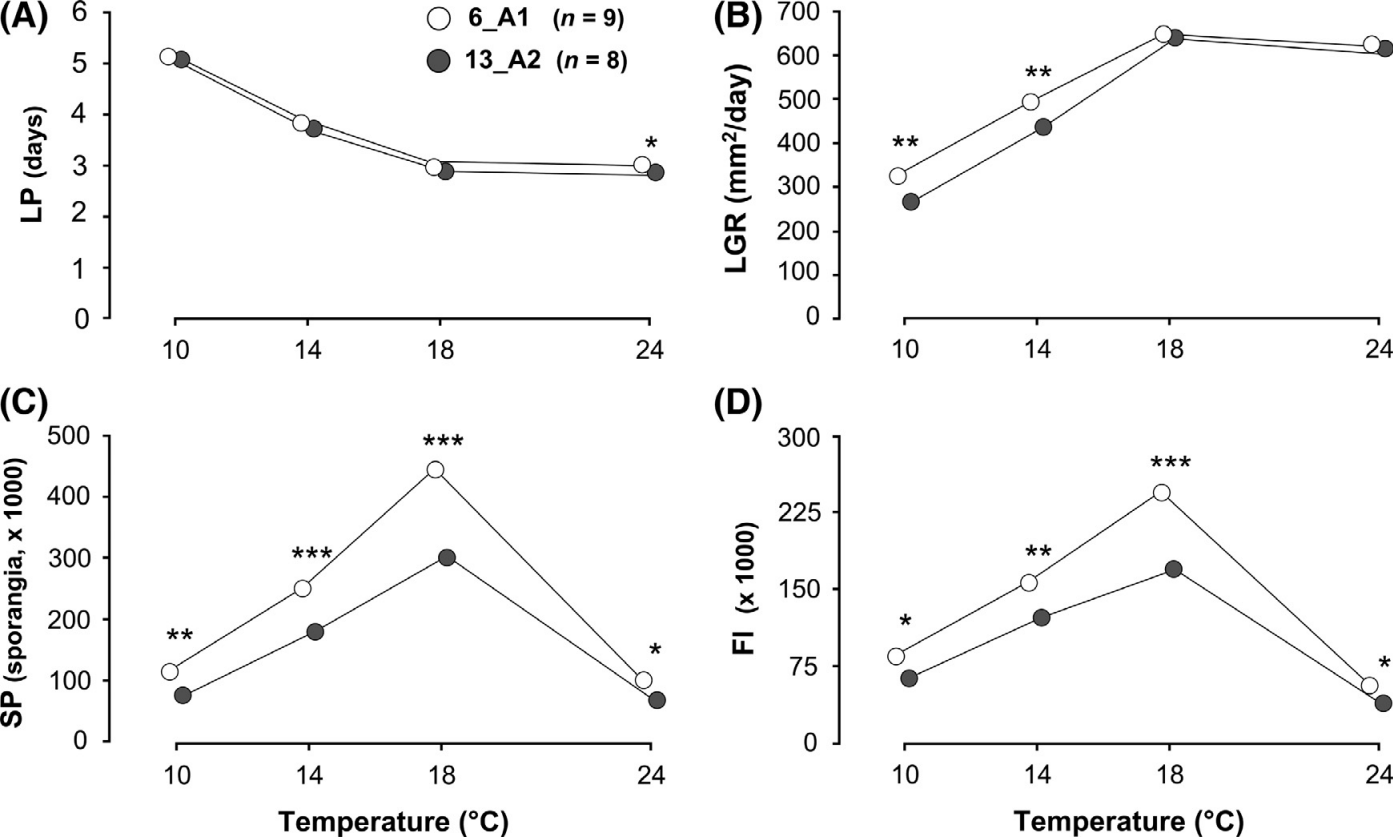
Temperature responses of 17 west European *Phytophthora infestans* isolates belonging to two clonal lineages (6_A1 and 13_A2) for latent period (A), lesion growth rate (B), sporangia production (C), and sporangia size (D). SE were omitted for clarity. Significant differences between the clonal lineages at a given temperature, as revealed by Wilcoxon rank‐sum tests or lsmeans: *P <* *0.05, **P <* *0.01, ***P <* *0.001.

Clonal lineages had strong effects on life‐history traits and on FI of west European isolates (Fig. 4; Table 4). 6_A1 isolates had a significantly higher LGR at 10 and 14°C (Wald's test, lsmeans post hoc comparison; *P *<* *0.01; Fig. 4B) as well as a significantly higher SP and FI at the four temperatures tested (Wald's test, lsmeans post hoc comparison; *P *<* *0.05–<0.001; Fig. 4C and D) than 13_A2 isolates collected in the same area at the same time.

**Table 4:** Linear mixed‐model analysis of the clonal lineage (6_A1 and 13_A2) and the temperature effect (10, 14, 18, and 24°C) on the lesion growth rate (LGR), the sporangia production (SP), and the fitness index (FI) of 17 *Phytophthora infestans* isolates sampled in western Europe

Response variable	LGR			SP			FI		
	df	Wald *χ*²	*P *> *χ*²	df	Wald *χ*²	*P *> *χ*²	df	Wald *χ*²	*P *> *χ*²
Clonal lineage	1	2.067	0.1506	1	13.291	<0.0001***	1	11.066	<0.0001***
Temperature	3	602.853	<0.0001***	3	490.047	<0.0001***	3	505.907	<0.0001***
Origin × temperature	3	4.373	0.2240	3	4.748	0.1912	3	5.222	0.1562
Origin × temperature × repetition	8	6.000	0.6472	8	6.989	0.5378	8	9.156	0.3293

Significance levels as follows: ***P <* *0.001.

## Discussion

### *Phytophthora infestans* is locally adapted to temperature, both at the population and at the clonal lineage levels

This common‐garden experiment involving a range of *P. infestans* isolates allowed to show the existence of patterns of local adaptation in this species. The first test for local adaptation is the “home vs. away” test, where the performance of a given population is compared between native and non‐native environments (Kawecki and Ebert 2004). Here, the shapes of thermal response curves were quite similar for all isolates tested, regardless of their area of origin. This explains why the “home vs. away” criterion could not reveal local adaptation patterns here. The other way of detecting local adaptation is through the “local vs. foreign” test, that is, by measuring higher performances within a given environment for a population native to this environment than for populations transplanted from other environments (Kawecki and Ebert 2004). Here, this criterion was fulfilled for SP and the FI, with the highest fitness for the Nordic isolates at 10°C and for the west European and Mediterranean isolates at 18 and 24°C, respectively. This indicates that *P. infestans* populations are adapted to temperature conditions prevailing in the areas where populations were sampled from. Such patterns have previously been reported in other fungal pathogens of crops, such as *Rhynchosporium commune* on barley (Stefansson et al. 2013) or *P. striiformis* on wheat (Mboup et al. 2012), but it is to our knowledge the first report of such a local adaptation to climate in *P. infestans*.

Our data also show that local adaptation to temperature can occur not only between populations, but also within a single clonal lineage. 13_A2 isolates indeed had different performances across the range of temperatures tested depending on the area of sampling. West European 13_A2 isolates caused larger lesions and produced more sporangia at low temperatures than their Mediterranean counterparts; the opposite was true at high temperatures. These observations, fulfilling the “local vs. foreign” criterion of local adaptation (Kawecki and Ebert 2004), are to our knowledge the first report of intraclonal local adaptation to temperature in any microbial pathogen.

### Sympatric clones show no “uniform” response to temperature

Local adaptation to temperature coupled with the similarity of response norms between isolates of *P. infestans* would lead to assume that sympatric isolates would share thermal response patterns, even if they belonged to different clones. However, this is not the case. We indeed showed that two sympatric clonal lineages, 6_A1 and 13_A2, had distinctly different performance over the whole range of temperatures tested. 6_A1 isolates had indeed significantly higher LGRs at low temperatures and produced more sporangia over the range of temperatures tested than 13_A2 isolates. Differences in thermal response between *P. infestans* clonal lineages have been reported previously, for example, in an experiment with Brazilian isolates where BR‐1 showed higher sporulation capabilities at low temperatures than US‐1, and conversely at high temperatures (Maziero et al. 2009). Other clonal lineages were found to have similar temperature responses, such as US‐1 and US‐8 (Miller and Johnson 2014). In contrast to American clones, little information was available on the temperature responses of the current European clonal lineages. Cooke et al. (2012) have nonetheless tested the effect of two temperatures on nine clonal lineages from Great Britain. They have shown that at 13°C, both 13_A2 and 6_A1 were part of those which caused the largest lesions, whereas at 18°C, 6_A1 isolates caused the largest lesions. These results do not match with ours, where 13_A2 isolates were found to perform badly at low temperatures. The lower LGRs and SP levels of 13_A2 isolates at low temperatures compared to 6_A1 isolates, as well as their shorter LP at 24°C, suggest a better adaptation of this lineage to warmer conditions. This could avoid the expansion of 13_A2 to cool climates, and therefore explain its absence from NE as hypothesized by Chmielarz et al. (2014).

### Is the response of life‐history traits to temperature a consequence of survival strategies?

If the low performances of the Nordic isolates were expected at high temperatures, the fact that they also had the longest LPs and the lowest LGR at low temperatures is more surprising. A maladaptation of these isolates to potato cv. Bintje used in our tests can be excluded, given the frequent and prolonged use of this cultivar in NE. We can thus suppose that Nordic isolates are selected for long LPs and low LGRs. Such traits would favor co‐inoculations by other isolates and therefore increase the probability of finding a sexual partner and produce oospores (Clément et al. 2012). Such “slow” isolates would benefit then from increased interepidemic transmission in NE, where the climatic conditions allow overwintering survival primarily through the sexual oospores. Nevertheless, this hypothesis implies a high heritability of phenotypic characteristics, which was not always observed in previous studies (Knapova et al. 2002; Klarfeld et al. 2009).

While adaptation to sexual overwintering might explain the temperature response patterns in Nordic isolates, adaptation to asexual survival could be the clue for temperature response patterns in west European and Mediterranean isolates. Climatic conditions can be relatively close in WE and in the MB when potato is grown: April to September in WE; December to May (spring crop) or September to January (winter crop) in the MB. The local adaptive patterns to temperature observed within the 13_A2 clonal lineage could thus be shaped by thermal differences between WE and the MB throughout the entire year, notably during the periods when the potato is not cultivated. In both areas, *P. infestans* is primarily transmitted asexually between cropping seasons of its host, mainly *via* volunteer or infected seed tubers (Andrivon 1995; Zwankhuizen et al. 2000). During this asexual survival stage, the pathogen can undergo severe climatic conditions, with low temperatures during west European winters or high temperatures during Mediterranean summers. This would lead to local adaptation to temperature, if selection occurs during survival rather than during epidemics. To confirm this hypothesis, it could be interesting to compare the survival of isolates from these two areas at extreme climatic conditions such as temperatures below 5°C or above 25°C, which can be frequent during west European winters and MB summers, respectively.

If local adaptation to temperature results from selection for increased survival between epidemics, it should influence the diffusion and invasion patterns by *P. infestans* lineages. For example, the 13_A2 lineage, first detected in the Netherlands and Germany in 2004 (Cooke et al. 2012), has rapidly replaced other clonal lineages to become dominant in WE within a few years, and it is still currently one of the more prevalent clonal lineages in this area (Euroblight.net). Its invasive success may appear surprising in view of its lower FI compared with 6_A1, as reported in this study. These results are especially due to lower performances of 13_A2 for LGR and SP, as both traits are directly involved in aggressiveness (i.e., the quantity of disease induced by a pathogenic strain on a susceptible host; Andrivon 1993) and thus thought to contribute to the epidemiologic fitness of *P. infestans* (Day and Shattock 1997). Several assumptions can be put forward to explain the high frequencies of 13_A2 isolates despite their low relative aggressiveness. First, although less aggressive than 6_A1, 13_A2 may be more aggressive than the other clonal lineages present in west European populations, as suggested by Cooke et al. (2012). This hypothesis was, however, contradicted by the phenotypic characterization of French isolates over an 8‐year period bridging the 13_A2 invasion, which revealed a lower aggressiveness of 13_A2 compared to all other prevalent clonal lineages, such as 8_A1 or 2_A1 (Mariette et al. 2016). A second possibility is that the low aggressiveness of 13_A2 isolates allows them to be better transmitted from epidemic to epidemic, and thus have a crucial advantage against other isolates. This trade‐off between intra‐epidemic transmission (i.e., aggressiveness) and interepidemic transmission has been recently demonstrated (Pasco et al. 2016). Finally, a trade‐off between the number of sporangia produced and their size could also be involved. 13_A2 isolates indeed produced fewer sporangia, but bigger ones than 6_A1 (data not shown), resulting in a probably lower fitness deficit than would be expected based on spore production alone, especially at low temperatures.

### High temperatures could be more penal than low temperatures to *P. infestans* fitness

We observed a strong effect of temperature on the three life‐history traits measured, for isolates of each population. The optimal temperature range for the development of *P. infestans* was reported between 15 and 22°C (Mizubuti and Fry 1998; Maziero et al. 2009; Shakya et al. 2015), which is in line with our results as the isolates had best performances at 18°C, especially for SP. This translates into a narrow range of temperatures of maximal fitness in *P. infestans* populations.

Although the conditions allowing optimum fitness are those problematic for the management of diseases, the response to the extreme parts of the temperature range allowing pathogen activity is also of great relevance. While *P. infestans* isolates did not perform well at either 10 or 24°C, confirming the restricted development of this species at low temperatures (Mizubuti and Fry 1998; Andrade‐Piedra et al. 2005), the FI values tended to be higher on the lower side of the temperature range than on the higher side of this range. This suggests that low temperatures might be less detrimental to *P. infestans* than higher temperatures, which also condition a lack of available free water needed for infection success. Our biological observations therefore support the predictions based on climate change models as to the stable or reduced risk of late blight and distribution of the disease in future decades (Launay et al. 2014; Sparks et al. 2014).

## Conflict of Interest

None declared.

## Supporting information


**Figure S1.** Temperature survey within the four climatic chambers used for the common‐garden experiments (10, 14, 18, 24°C).

**Table S1.** Areas of sampling of the *Phytophthora infestans* isolates with their geographic characteristics (region, country, locations and coordinates) and climatic characteristics (MAT, the mean annual temperature in °C; Min and Max, the minimum and maximum monthly mean temperatures; varMAT, the variance in mean annual temperature).
**Table S2.** Characteristics of the isolates of *Phytophthora infestans* used in the experiments with the country and the region of origin, the mating type and the clonal lineage of belonging.

## References

[ece32282-bib-0001] Andrade‐Piedra, J. L. , R. J. Hijmans , G. A. Forbes , W. E. Fry , and R. J. Nelson . 2005 Simulation of potato late blight in the Andes. I: modification and parameterization of the LATEBLIGHT model. Phytopathology 95:1191–1199. 18943472 10.1094/PHYTO-95-1191

[ece32282-bib-0002] Andrivon, D. 1993 Nomenclature for pathogenicity and virulence: the need for precision. Phytopathology 83:889–890.

[ece32282-bib-0003] Andrivon, D. 1995 Biology, ecology and epidemiology of the potato late blight pathogen *Phytophthora infestans* in soil. Phytopathology 85:1053–1056.

[ece32282-bib-0004] Andrivon, D. , J. Montarry , R. Corbière , C. Pasco , I. Glais , B. Marquer , et al. 2013 The hard life of *Phytophthora infestans*: when trade‐offs shape evolution in a biotrophic plant pathogen. Plant. Pathol. 62(Suppl. 1):28–35.

[ece32282-bib-0005] Aylor, D. E. 2003 Spread of plant disease on a continental scale: role of aerial dispersal of pathogens. Ecology 84:1989–1997.

[ece32282-bib-0006] Bates, D. , M. Maechler , and B. Bolker . 2011 lme4: Linear mixed‐effects models using S4 classes. R package version 0.999375‐39. Available at http://CRAN.R-project.org/package=lme4. (accessed 15 March 2016).

[ece32282-bib-0007] Bebber, D. P. , M. A. T. Ramotowski , and S. J. Gurr . 2013 Crop pest and pathogens move polewards in a warming world. Nat. Clim. Chang. 3:985–988.

[ece32282-bib-0008] Bellard, C. , C. Bertelsmeier , P. Leadley , W. Thuiller , and F. Courchamp . 2012 Impacts of climate change on the future of biodiversity. Ecol. Lett. 15:365–377. 22257223 10.1111/j.1461-0248.2011.01736.x PMC3880584

[ece32282-bib-0009] Bennett, A. F. , and R. E. Lenski . 1999 Experimental evolution and its role in evolutionary physiology. Am. Zool. 39:346–362.

[ece32282-bib-0010] Blanquart, F. , O. Kaltz , S. Nuismer , and S. Gandon . 2013 A practical guide to measuring local adaptation. Ecol. Lett. 16:1195–1205. 23848550 10.1111/ele.12150

[ece32282-bib-0011] Boenigk, J. , S. Jost , T. Stoeck , and T. Garstecki . 2007 Differential thermal adaptation of clonal strains of a protist morphospecies originating from different climatic zones. Environ. Microbiol. 9:593–602. 17298360 10.1111/j.1462-2920.2006.01175.x

[ece32282-bib-0012] Chmielarz, M. , S. Sobkowiak , K. Dębski , D. E. L. Cooke , M. B. Brurberg , and J. Śliwka . 2014 Diversity of *Phytophthora infestans* from Poland. Plant. Pathol. 63:203–211.

[ece32282-bib-0013] Chowdappa, P. , B. J. N. Kumar , S. Madhura , S. P. M. Kumar , K. L. Myers , W. E. Fry , et al. 2015 Severe outbreaks of late blight on potato and tomato in South India caused by recent changes in the *Phytophthora infestans* population. Plant. Pathol. 64:191–199.

[ece32282-bib-0014] Clément, J. A. J. , H. Magalon , I. Glais , E. Jacquot , and D. Andrivon . 2012 To be or not to be solidary: *Phytophthora infestans*’ dilemma for optimizing its reproductive fitness in intra‐ and intergenotype multiple infections. PLoS One 7:e37838. 22675493 10.1371/journal.pone.0037838 PMC3365895

[ece32282-bib-0015] Cooke, D. E. L. , L. M. Cano , S. R. Raffaele , R. A. Bain , L. R. Cooke , G. J. Etherington , et al. 2012 Genome analyses of an aggressive and invasive lineage of the Irish potato famine pathogen. PLoS Pathog. 8:e1002940. 23055926 10.1371/journal.ppat.1002940 PMC3464212

[ece32282-bib-0016] Corbière, R. , F. Z. Rekad , A. Galfout , D. Andrivon , and Z. Bouznard . 2010 Phenotypic and genotypic characteristics of Algerian isolates of Phytophthora infestans. Proc. 12th Euroblight Workshop, Arras, France. PPO Spec. Rep. No. 14, 133–146.

[ece32282-bib-0017] Cossins, A. R. , and K. Bowler . 1987 Temperature biology of animals. Chapman & Hall, New York.

[ece32282-bib-0018] Day, J. P. , and R. C. Shattock . 1997 Aggressiveness and other factors relating to displacement of populations of *Phytophthora infestans* in England and Wales. Eur. J. Plant Pathol. 103:379–391.

[ece32282-bib-0019] van Doorslaer, W. , J. Vanoverbeke , C. Duvivier , S. Rousseaux , M. Jansen , B. Jansen , et al. 2009 Local adaptation to higher temperatures reduces immigration success of genotypes from a warmer region in the water flea *Daphnia* . Glob. Change Biol. 15:3046–3055.

[ece32282-bib-0020] Drenth, A. , E. M. Janssen , and F. Govers . 1995 Formation and survival of oospores of *Phytophthora infestans* . Plant. Pathol. 44:86–94.

[ece32282-bib-0021] Gisi, U. , F. Walder , Z. Resheat‐Eini , D. Edel , and H. Sierotzki . 2011 Changes of genotype, sensitivity and aggressiveness in *Phytophthora infestans* isolates collected in European countries in 1997, 2006 and 2007. J. Phytopathol. 159:223–232.

[ece32282-bib-0022] Glais, I. , J. Montarry , R. Corbière , C. Pasco , B. Marquer , H. Magalon , et al. 2014 Long‐distance gene flow outweighs a century of local selection and prevents local adaptation in the Irish famine pathogen *Phytophthora infestans* . Evol. Appl. 7:442–452. 24822079 10.1111/eva.12142 PMC4001443

[ece32282-bib-0023] Goss, E. M. , J. F. Tabima , D. E. L. Cooke , S. Restrepo , W. E. Fry , G. A. Forbes , et al. 2014 The Irish potato famine pathogen *Phytophthora infestans* originated in central Mexico rather than the Andes. Proc. Natl Acad. Sci. USA 111:8791–8796. 24889615 10.1073/pnas.1401884111 PMC4066499

[ece32282-bib-0024] Harbaoui, K. , W. Hamada , Y. Li , V. G. A. A. Vleeshouwers , and T. van der Lee . 2014 Increased difficulties to control late blight in Tunisia are caused by a genetically diverse *Phytophthora infestans* population next to the clonal lineage NA‐01. Plant Dis. 98:898–908. 10.1094/PDIS-06-13-0610-RE 30708842

[ece32282-bib-0025] Hochachka, P. W. , and G. N. Somero . 2002 Biochemical adaptation: mechanism and process in physiological evolution. Oxford Univ. Press, New York.

[ece32282-bib-0026] Hoffmann, A. A. , and C. M. Sgrò . 2011 Climate change and evolutionary adaptation. Nature 470:479–485. 21350480 10.1038/nature09670

[ece32282-bib-0027] IPCC . (2014) Climate change 2014: synthesis report 151 Pp. *in* Core Writing Team , Pachauri R. K., Meyer L. A., eds. Contribution of working groups I, II and III to the fifth assessment report of the intergovernmental panel on climate change. IPCC, Geneva, Switzerland.

[ece32282-bib-0028] Jinks, J. L. , and M. Grindle . 1963 Changes induced by training in *Phytophthora infestans* . Heredity 18:245–264.

[ece32282-bib-0029] Kaltz, O. , and J. A. Shykoff . 1998 Local adaptation in host‐parasite systems. Heredity 81:361–370.

[ece32282-bib-0030] Kamoun, S. , O. Furzer , J. D. J. Jones , H. S. Judelson , G. S. Ali , R. J. D. Dalio , et al. 2015 The Top 10 oomycete pathogens in molecular plant pathology. Mol. Plant Pathol. 16:413–434. 25178392 10.1111/mpp.12190 PMC6638381

[ece32282-bib-0031] Kawecki, T. J. , and D. Ebert . 2004 Conceptual issues in local adaptation. Ecol. Lett. 7:1225–1241.

[ece32282-bib-0032] Klarfeld, S. , A. E. Rubin , and Y. Cohen . 2009 Pathogenic fitness of oosporic progeny isolates of *Phytophthora infestans* on late‐blight‐resistant tomato lines. Plant Dis. 93:947–953. 10.1094/PDIS-93-9-0947 30754538

[ece32282-bib-0033] Knapova, G. , and U. Gisi . 2002 Phenotypic and genotypic structure of *Phytophthora infestans* populations on potato and tomato in France and Switzerland. Plant. Pathol. 51:641–653.

[ece32282-bib-0034] Knapova, G. , A. Schlenzig , and U. Gisi . 2002 Crosses between isolates of *Phytophthora infestans* from potato and tomato and characterization of F‐1 and F‐2 progeny for phenotypic and molecular markers. Plant. Pathol. 51:698–709.

[ece32282-bib-0035] Launay, M. , J. Caubel , G. Bourgeois , F. Huard , I. G. de Cortazar‐Atauri , M. O. Bancal , et al. 2014 Climatic indicators for crop infection risk: application to climate change impacts on five major foliar fungal diseases in Northern France. Agric. Ecosyst. Environ. 197:147–158.

[ece32282-bib-0036] Lees, A. K. , R. Wattier , D. S. Shaw , L. Sullivan , N. A. Williams , and D. E. L. Cooke . 2006 Novel microsatellite markers for the analysis of *Phytophthora infestans* populations. Plant. Pathol. 55:311–319.

[ece32282-bib-0037] Li, Y. , D. E. L. Cooke , E. Jacobsen , and T. van der Lee . 2013a Efficient multiplex simple sequence repeat genotyping of the oomycete plant pathogen *Phytophthora infestans* . J. Microbiol. Methods 92:316–322. 23313554 10.1016/j.mimet.2012.11.021

[ece32282-bib-0038] Li, Y. , T. van der Lee , J. Zhu , et al. 2013b Population structure of *Phytophthora infestans* in China – geographic clusters and presence of the EU genotype Blue‐13. Plant. Pathol. 62:932–942.

[ece32282-bib-0039] Lively, C. M. 1996 Host‐parasite coevolution and sex. Bioscience 46:107–114.

[ece32282-bib-0040] Mariette, N. , R. Mabon , R. Corbière , F. Boulard , I. Glais , B. Marquer , et al. 2016 Phenotypic and genotypic changes in French populations of *Phytophthora infestans*: are invasive clones the most aggressive? Plant. Pathol. 65:577–586.

[ece32282-bib-0041] Maziero, J. M. N. , L. A. Maffia , and E. S. G. Mizubuti . 2009 Effects of temperature on events in the infection cycle of two clonal lineages of *Phytophthora infestans* causing late blight on tomato and potato in Brazil. Plant Dis. 93:459–466. 10.1094/PDIS-93-5-0459 30764133

[ece32282-bib-0042] Mboup, M. , B. Bahri , M. Leconte , C. De Vallavieille‐Pope , O. Kaltz , and J. Enjalbert . 2012 Genetic structure and local adaptation of European wheat yellow rust populations: the role of temperature‐specific adaptation. Evol. Appl. 5:341–352. 25568055 10.1111/j.1752-4571.2011.00228.x PMC3353355

[ece32282-bib-0043] Miller, J. S. , and D. A. Johnson . 2014 Aggressiveness of *Phytophthora infestans* genotypes on potato stems and leaves at three temperatures. Am. J. Potato Res. 91:538–553.

[ece32282-bib-0044] Mitchell, S. E. , and W. Lampert . 2000 Temperature adaptation in a geographically widespread zooplankter, *Daphnia magna* . J. Evol. Biol. 13:371–382.

[ece32282-bib-0045] Mizubuti, E. G. S. , and W. E. Fry . 1998 Temperature effects on developmental stages of isolates from three clonal lineages of *Phytophthora infestans* . Phytopathology 88:837–843. 18944891 10.1094/PHYTO.1998.88.8.837

[ece32282-bib-0046] Montarry, J. , I. Glais , R. Corbiere , and D. Andrivon . 2008 Adaptation to the most abundant host genotype in an agricultural plant‐pathogen system – potato late blight. J. Evol. Biol. 21:1397–1407. 18547352 10.1111/j.1420-9101.2008.01557.x

[ece32282-bib-0047] Montarry, J. , D. Andrivon , I. Glais , G. Mialdea , R. Corbière , and F. Delmotte . 2010a Microsatellite markers reveal two genetic groups in the French population of the invasive plant pathogen *Phytophthora infestans* . Mol. Ecol. 19:1965–1977. 20345671 10.1111/j.1365-294X.2010.04619.x

[ece32282-bib-0048] Montarry, J. , F. M. Hamelin , I. Glais , R. Corbière , and D. Andrivon . 2010b Fitness costs associated with unnecessary virulence factors and life history traits: evolutionary insights from the potato late blight pathogen *Phytophthora infestans* . BMC Evol. Biol. 10:283. 20846405 10.1186/1471-2148-10-283 PMC2949872

[ece32282-bib-0049] Parmesan, C. 2006 Ecological and evolutionary responses to recent climate change. Annu. Rev. Ecol. Evol. Syst. 37:637–669.

[ece32282-bib-0050] Pasco, C. , J. Montarry , B. Marquer , and D. Andrivon . 2016 And the nasty ones lose in the end: foliar pathogenicity trades off with asexual transmission in the Irish famine pathogen *Phytophthora infestans* . New Phytol. 209:334–342. 26295446 10.1111/nph.13581

[ece32282-bib-0051] R Core Team . 2014 R: a language and environment for statistical computing. R Foundation for Statistical Computing, Vienna, Austria.

[ece32282-bib-0052] Rengefors, K. , R. Logares , J. Laybourn‐Parry , and R. J. Gast . 2015 Evidence of concurrent local adaptation and high phenotypic plasticity in a polar microeukaryote. Environ. Microbiol. 17:1510–1519. 25041758 10.1111/1462-2920.12571

[ece32282-bib-0053] Runno‐Paurson, E. , R. Kiiker , T. Joutsjoki , and A. Hannukkala . 2016 High genotypic diversity found among population of *Phytophthora infestans* collected in Estonia. Fungal Biol. 120:385–392. 26895867 10.1016/j.funbio.2015.11.008

[ece32282-bib-0054] Shakya, S. K. , E. M. Goss , N. S. Dufault , and A. H. C. van Bruggen . 2015 Potential effects of diurnal temperature oscillations on potato late blight with special reference to climate change. Phytopathology 105:230–238. 25140388 10.1094/PHYTO-05-14-0132-R

[ece32282-bib-0055] Sharma, K. , B. D. Gossen , and M. R. McDonald . 2011 Effect of temperature on cortical infection by *Plasmodiophora brassicae* and clubroot severity. Phytopathology 101:1424–1432. 21864086 10.1094/PHYTO-04-11-0124

[ece32282-bib-0056] Sjöholm, L. , B. Andersson , N. Högberg , A. K. Widmark , and J. Yuen . 2013 Genotypic diversity and migration patterns of *Phytophthora infestans* in the Nordic countries. Fungal Biol. 117:722–730. 24119411 10.1016/j.funbio.2013.08.002

[ece32282-bib-0057] Sparks, A. H. , G. A. Forbes , R. J. Hijmans , and K. A. Garrett . 2014 Climate change may have limited effect on global risk of potato late blight. Glob. Change Biol. 20:3621–3631. 10.1111/gcb.12587 24687916

[ece32282-bib-0058] Stefansson, T. S. , B. A. McDonald , and Y. Willi . 2013 Local adaptation and evolutionary potential along a temperature gradient in the fungal pathogen *Rhynchosporium commune* . Evol. Appl. 6:524–534. 23745143 10.1111/eva.12039 PMC3673479

[ece32282-bib-0059] Tooley, P. W. , M. Browning , K. L. Kyde , and D. Berner . 2009 Effect of temperature and moisture period on infection of *Rhododendron* ‘Cunningham's White’ by *Phytophthora ramorum* . Phytopathology 99:1045–1052. 19671006 10.1094/PHYTO-99-9-1045

[ece32282-bib-0060] Weisse, T. , T. Berendonk , N. Kamjunke , M. Moser , U. Scheffel , P. Stadler , et al. 2011 Significant habitat effects influence protist fitness: evidence for local adaptation from acidic mining lakes. Ecosphere 2:art134.

[ece32282-bib-0061] Zhan, J. , and B. A. McDonald . 2011 Thermal adaptation in the fungal pathogen *Mycosphaerella graminicola* . Mol. Ecol. 20:1689–1701. 21395890 10.1111/j.1365-294X.2011.05023.x

[ece32282-bib-0062] Zwankhuizen, M. J. , F. Govers , and J. C. Zadoks . 2000 Inoculum sources and genotypic diversity of *Phytophthora infestans* in Southern Flevoland, the Netherlands. Eur. J. Plant Pathol. 106:667–680.

