# Supplementary figures and images for: Local adaptation to temperature in populations and clonal lineages of the Irish potato famine pathogen Phytophthora infestans
Source: Ecol Evol. 2016 Aug 14;6(17):6320–31. doi: 10.1002/ece3.2282 (PMC5016652; doi:10.1002/ece3.2282)

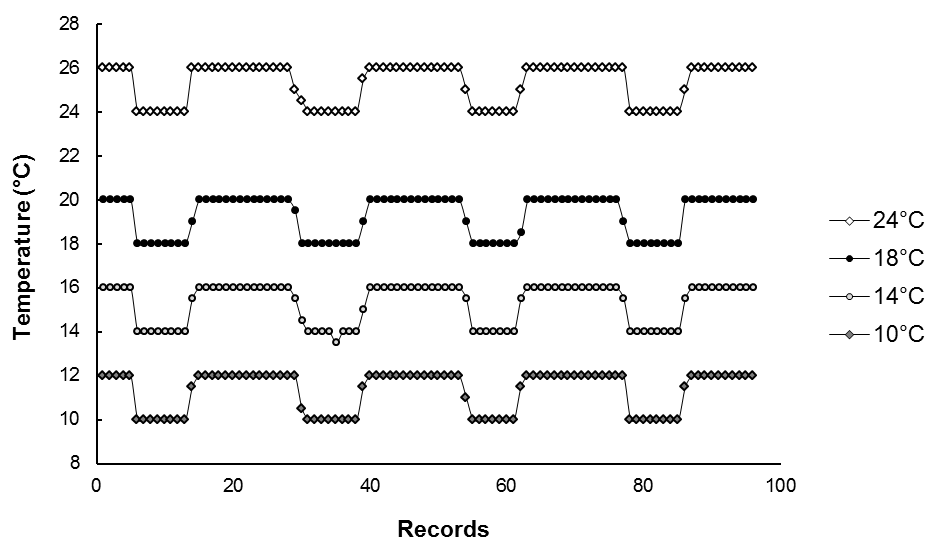

Supplement: Supplementary file 1 — Figure S1. Temperature survey within the four climatic chambers used for the common‐garden experiments (10, 14, 18, 24°C). [file ECE3-6-6320-s001.png]
