# Supplementary material for: Local adaptation to temperature in populations and clonal lineages of the Irish potato famine pathogen Phytophthora infestans
Source: Ecol Evol. 2016 Aug 14;6(17):6320–31. doi: 10.1002/ece3.2282 (PMC5016652; doi:10.1002/ece3.2282)
Supplement: Supplementary file 2 — Table S1. Areas of sampling of the Phytophthora infestans isolates with their geographic characteristics (region, country, locations and coordinates) and climatic characteristics (MAT, the mean annual temperature in °C; Min and Max, the minimum and maximum monthly mean temperatures; varMAT, the variance in mean annual temperature). Table S2. Characteristics of the isolates of Phytophthora infestans used in the experiments with the country and the region of origin, the mating type and the clonal lineage of belonging. [file ECE3-6-6320-s002.docx]

**Supporting information**

Fig. S1. Temperature survey within the four climatic chambers used for the common-garden experiments (10°C, 14°C, 18°C, 24°C). The records were made every hour during four consecutive days, from 03/10/14 to 03/13/14 (i.e. 96 records), using Thermo Tracer recorders (Oceasoft, Montpellier, France). This tracking reveals an increase of the temperature by 2°C during the diurnal phase compared to the initial parameters of the chambers.

**Table S1.** Areas of sampling of the *Phytophthora infestans* isolates with their geographic characteristics (region, country, locations and coordinates) and climatic characteristics (MAT, the mean annual temperature in °C; Min and Max, the minimum and maximum monthly mean temperatures; varMAT, the variance in mean annual temperature). This weather data was collected between 1982 and 2012 (http://climate-data.org).

| **Region** | **Country** | **Location** | **Latitude** | **Longitude** | **MAT** | **Min** | **Max** | **varMAT** |
| --- | --- | --- | --- | --- | --- | --- | --- | --- |
| Northern Europe | Denmark | Aarhus | 56.15°N | 10.22°E | 7.8 | -0.7 | 16.4 | 40.0 |
|  | Estonia | Tartu | 58.38°N | 26.72°E | 5.0 | -7.0 | 16.9 | 80.5 |
|  | Latvia | Valmiera | 57.55°N | 25.40°E | 5.5 | -6.2 | 16.9 | 75.6 |
|  | Lithuania | Kaunas | 54.89°N | 23.89°E | 6.6 | -5.1 | 17.3 | 71.4 |
| Western Europe | France | Paimpol | 48.78°N | 3.05°W | 11.5 | 5.8 | 17.5 | 20.2 |
| Mediterranean Basin | Algeria | Djendel | 36.22°N | 2.41°E | 18.0 | 9.7 | 27.8 | 45.1 |
|  | Cyprus | Xylofagou | 34.98°N | 33.85°E | 19.1 | 13.6 | 24.7 | 36.9 |

**Table S2.** Characteristics of the isolates of *Phytophthora infestans* used in the experiments with the country and the region of origin, the mating type and the clonal lineage of belonging.

| **Isolat** | **Country** | **Region** | **Mating type** | **Clonal lineage^a^** |
| --- | --- | --- | --- | --- |
| FR.13.P10.11 | France | Western Europe | A2 | 13_A2 |
| FR.13.P11.06 | France | Western Europe | A2 | 13_A2 |
| FR.13.P11.17 | France | Western Europe | A2 | 13_A2 |
| FR.13.P12.10 | France | Western Europe | A2 | 13_A2 |
| FR.13.P12.25 | France | Western Europe | A2 | 13_A2 |
| FR.13.P12.26 | France | Western Europe | A1 | 6_A1 |
| FR.13.P14.13 | France | Western Europe | A1 | 6_A1 |
| FR.13.P15.03 | France | Western Europe | A2 | 13_A2 |
| FR.13.P17.14 | France | Western Europe | A1 | 6_A1 |
| FR.13.P17.15 | France | Western Europe | A1 | 6_A1 |
| FR.13.P17.17A | France | Western Europe | A2 | 13_A2 |
| FR.13.P18.05 | France | Western Europe | A1 | 6_A1 |
| FR.13.P18.08 | France | Western Europe | A1 | 6_A1 |
| FR.13.P19.03 | France | Western Europe | A1 | 6_A1 |
| FR.13.P20.08 | France | Western Europe | A1 | 6_A1 |
| FR.13.P20.12 | France | Western Europe | A1 | 6_A1 |
| FR.13.P20.19 | France | Western Europe | A2 | 13_A2 |
|  |  |  |  |  |
| CY.13.01B | Cyprus | Mediterranean Basin | A2 | 13_A2 |
| CY.13.04B | Cyprus | Mediterranean Basin | A2 | 13_A2 |
| CY.13.09 | Cyprus | Mediterranean Basin | A2 | 13_A2 |
| CY.13.14 | Cyprus | Mediterranean Basin | A2 | 13_A2 |
| DZ.12.P01 | Algeria | Mediterranean Basin | A2 | NA |
| DZ.12.P02 | Algeria | Mediterranean Basin | A2 | NA |
| DZ.13.P01 | Algeria | Mediterranean Basin | A1 | 2A1 |
| DZ.13.P03 | Algeria | Mediterranean Basin | A2 | 13_A2 |
| DZ.13.P05 | Algeria | Mediterranean Basin | A2 | 13_A2 |
|  |  |  |  |  |
| DA.13.8.37 | Denmark | Northern Europe | A2 | NA |
| DA.13.8.41 | Denmark | Northern Europe | A2 | NA |
| DA.13.8.47 | Denmark | Northern Europe | A2 | NA |
| DA.13.8.50 | Denmark | Northern Europe | A1 | NA |
| LT.AK24.13 | Lithuania | Northern Europe | A1 | NA |
| LT.SA1.13 | Lithuania | Northern Europe | A2 | NA |
| LT.SI.1.13 | Lithuania | Northern Europe | A1 | NA |
| LT.ST2.13 | Lithuania | Northern Europe | A1 | NA |
| LV.BI7.13 | Latvia | Northern Europe | A2 | NA |
| LV.GO12.13 | Latvia | Northern Europe | A2 | NA |
| LV.JA7.13 | Latvia | Northern Europe | A2 | NA |
| LV.RA4.13 | Latvia | Northern Europe | A1 | NA |
| EST.RE3.13 | Estonia | Northern Europe | A1 | NA |
| EST.SA.AS7.13 | Estonia | Northern Europe | A2 | NA |
| EST.VE3.13 | Estonia | Northern Europe | A1 | NA |
| EST.JO(I).4.13 | Estonia | Northern Europe | A1 | NA |

**^a^** Clonal lineages were determined by comparing the isolates fingerprints with those described by Cooke *et al*. (2012). NA signifies a non-belonging to a referenced clonal lineage.
